# Comprehensive analyses of a CD8^+^ T cell infiltration related gene signature with regard to the prediction of prognosis and immunotherapy response in lung squamous cell carcinoma

**DOI:** 10.1186/s12859-023-05302-3

**Published:** 2023-06-06

**Authors:** Liang Chen, Yiming Weng, Xue Cui, Qian Li, Min Peng, Qibin Song

**Affiliations:** Cancer Center, Renmin Hospital of Wuhan University, Wuhan, 430060 China

**Keywords:** Lung squamous cell carcinoma, CD8^+^ T cell, Prognosis, Immunotherapy

## Abstract

Lung squamous cell carcinoma (LUSC) is associated with a worse prognosis than other histological subtypes of non-small cell lung cancer. Due to the vital role of CD8^+^ T cells in anti-tumor immunity, the characterization of CD8^+^ T cell infiltration-related (CTLIR) gene signature in LUSC is worthy of in-depth exploration. In our study, tumor tissues of LUSC patients from Renmin Hospital of Wuhan University were stained by multiplex immunohistochemistry to evaluate the density of infiltrated CD8^+^ T cells and explore the correlation with immunotherapy response. We found that the proportion of LUSC patients who responded to immunotherapy was higher in the high density of CD8^+^ T cell infiltration group than in the low density of CD8^+^ T cell infiltration group. Subsequently, we collected bulk RNA-sequencing data from The Cancer Genome Atlas (TCGA) database. The abundance of infiltrating immune cells in LUSC patients was analyzed by using CIBERSORT algorithm, and weighted correlation network analysis was performed to identify the co-expressed gene modules related to CD8^+^ T cells. We then developed a prognostic gene signature based on CD8^+^ T cell co-expressed genes and calculated the CTLIR risk score, which stratified LUSC patients into high-risk and low-risk groups. With univariate and multivariate analyses, the gene signature was identified as an independent prognostic factor in LUSC patients. The overall survival of LUSC patients in the high-risk group was significantly shorter than that of the low-risk group in the TCGA cohort, which was validated in Gene Expression Omnibus datasets. We analyzed immune cell infiltration in the tumor microenviroment and found fewer CD8^+^ T cells and more regulatory T cell infiltration in the high-risk group, which is characterized as an immunosuppressive phenotype. Furthermore, the LUSC patients in the high-risk group were predicted to have a better response to immunotherapy than those in the low-risk group when treated with PD-1 and CTLA4 inhibitors. In conclusion, we performed a comprehensive molecular analysis of the CTLIR gene signature in LUSC and constructed a risk model for LUSC patients to predict prognosis and immunotherapy response.

**Supplementary Information:**

The online version contains supplementary material available at 10.1186/s12859-023-05302-3.

## Introduction

Over the past few decades, lung cancer has been the leading cause of cancer death worldwide [1]. Lung squamous cell carcinoma (LUSC), which is a distinct histological subtype of non-small cell lung cancer (NSCLC), accounts for approximately 30% of all lung cancer cases [2]. Despite the rapid development of therapeutic approaches, the prognosis of LUSC remains poor, and the 5-year overall survival (OS) remains below 20% [3]. The efficacy of conventional treatment approaches, which include surgical resection, chemotherapy, and radiotherapy, to improve the prognosis of LUSC has reached its limits [4]. Additionally, oncogenic mutations and alterations approved for targeted therapies rarely occur in LUSC [5, 6].

Recently, immunotherapy has become a promising therapeutic strategy for LUSC [7]. Tumor cells exhibit various mechanisms to evade tumor immunosurveillance and suppress anti-tumor immune responses during cancer development and progression. Dysfunction of immune checkpoint molecules is a major mechanism underlying tumor immune evasion. Immune checkpoint blockade (ICB) helps the immune system recognize and attack tumor cells [8]. Immune checkpoint inhibitors targeting PD-1/PD-L1, and CTLA-4 have been approved for the treatment of advanced LUSC [9]. Although ICB has revolutionized cancer therapeutics, they are not effective for all patients. Among the cancer types for which ICB has proven efficacy, potent and durable response has been limited to a subgroup of patients, with several patients demonstrating a lack of initial response to the treatment of ICB. In fact, only one-third of patients respond to ICB in most cancer types [10]. Previous studies have shown that the objective response rate (ORR) to pembrolizumab is approximately 20% in advanced NSCLC [11, 12]. Several biological prognostic and predictive factors have been identified; however, no single biomarker can perfectly discriminate between responders and non-responders. Therefore, there is an urgent need to develop biological prognostic and predictive factors to identify patients who are most likely to respond to ICB therapy.

Various types of innate and adaptive immune cells reside within or infiltrate the tumor microenvironment [13]. The dynamic crosstalk between these immune cells and tumor cells defines the immune status of the tumor and can promote or hinder the tumor response to ICB [14]. CD8^+^ T cells play a central role in mediating anti-tumor immunity and eliminating tumor cells by recognizing tumor-associated antigens present in major histocompatibility complex class I [15]. CD8^+^ effector T cells exert an anti-tumor immune response through the release of cytolytic factors and the induction of apoptosis in tumor cells. The presence of CD8^+^ T cells at tumor margins and within the tumor prior to treatment with checkpoint inhibitors is associated with a stronger response to immunotherapy [16]. Increasing evidence has implicated the effect of CD8^+^ T cell infiltration on tumor prognosis [17]. Studies have shown that the infiltration of CD8^+^ T cells correlates with better prognosis in lung cancer [18]. Based on the abundance of CD4^+^ and CD8^+^ T cells and their penetration into the tumor, tumor immune profiles can be classified into “cold” or “hot” tumors or more precisely into the immune-inflamed phenotype, the immune-excluded phenotype, or the immune-desert phenotype, which are different in immunotherapy response [19]. Therefore, it is important to clarify the characteristics of the CD8^+^ T cell infiltration-related (CTLIR) gene signature in LUSC to provide a guidance for the administration of immunotherapy.

Herein, we identified the co-expression genes related to CD8^+^ T cells infiltration through weighted correlation network analysis (WGCNA) and established a risk model as a robust prognostic biomarker and predictive factor for immunotherapy response to enable informed decision-making.

## Materials and methods

### Multiplex immunohistochemistry (mIHC) of tumor tissues from LUSC patients

**Fig. 1:**
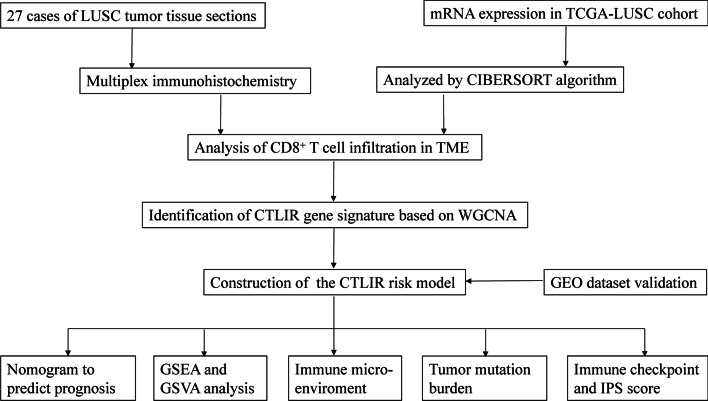
Flow chart of the study design. *LUSC* lung squamous cell carcinoma, *TCGA* The Cancer Genome Atlas, *TME* tumor microenvironment, *CTLIR* CD8^+^ T cell infiltration-related, *WGCNA* weighted correlation network analysis, *GEO* Gene Expression Omnibus, *GSEA* gene set enrichment analysis, *GSVA* gene set variation analysis, *IPS* immunophenoscore

An overview of the study design is shown in Fig. 1. A total of 27 LUSC samples with pathological sections were collected from the tissue bank in Renmin Hospital of Wuhan University. Formalin-fixed paraffin-embedded blocks were retrieved, and 4 µm-thick sections were prepared for mIHC staining. The tissue sections were immunostained using the PerkinElmer OPAL 7-Colour Manual IHC kit (NEL811001KT) and the following anti-human antibodies: CD8 (Dako, 1:100) and cytokeratin (CK, Dako, 1:200). Cell nuclei were stained with DAPI. Tumor islets and stroma were distinguished using CK staining. Briefly, tissue sections were baked at 65 °C for 1 h, followed by deparaffinization cycles. Microwave treatment was performed for antigen retrieval with EDTA buffer (pH 9.0). The tissue sections were blocked with antibody-blocking buffer for 12 min at room temperature. The sections were then incubated with primary antibodies overnight at 4 °C and then with secondary antibodies for 10 min at room temperature. Tyramide signal amplification was performed using Opal TSA Plus (1:10). Multiplex staining was performed by repeating the staining steps in a series. Fluorescent sections were scanned using the Vectra Polaris 3.0 (Akoya Biosciences, Marlborough, MA, United States of America) with 40 × magnification (Plan APO 40 × /NA 0.75, 0.25 µm/pixel) and auto-estimated exposure times [20]. The study was approved by the Ethics Committee of Renmin Hospital of Wuhan University (protocol code: WDRY2022-K041) on May 23, 2022.

### Data collection from public databases

Gene expression profiles and clinical data of LUSC patients were downloaded from the Genomic Data Commons Data Portal (https://portal.gdc.cancer.gov/) and Gene Expression Omnibus (GEO) (https://www.ncbi.nlm.nih.gov/geo/) databases. For The Cancer Genome Atlas (TCGA)-LUSC cohort, fragments per kilobase million values were transformed into transcripts per million values. Two expression datasets (GSE30219 and GSE37745) were downloaded from the GEO database. The gene expression information and clinical data of LUSC patients in these datasets were merged and then normalized using the “sva” R package. Only patients with complete relevant information were included in the study. The basic information of LUSC patients from The Cancer Genome Atlas (TCGA) and GEO databases in this study is documented in Additional file 5: Table S1 and Additional file 6: Table S2.

### Analysis of the abundance of infiltrated immune cells

CIBERSORT is an approach for characterizing cell subsets of interest in high-dimensional genomic data derived from bulk tissue samples and can estimate the proportions of 22 distinct immune cell types with gene expression profiles [21]. In this study, we analyzed the abundance of tumor-infiltrating immune cells in LUSC using the CIBERSORT algorithm.

### Identification of CTLIR gene signature based on weighted correlation network analysis (WGCNA)

WGCNA can be used to identify modules of highly correlated genes, summarize such clusters using the module eigengene (ME) or an intramodular hub gene, relate modules to one another and external sample traits using eigengene network methodology, and calculate module membership measures [22]. In our study, WGCNA was used to identify co-expressed gene modules closely related to multiple types of immune cells. Key modules were determined by calculating the Pearson’s correlation coefficient and *P* values of ME and immune cell types. We constructed a gene network and identified modules using the one-step network construction function of the “WGCNA” R package.

### Construction of the CTLIR risk model

Univariate Cox regression analysis was used to identify the prognostic value of the genes in a specific module. Least Absolute Shrinkage and Selection Operator (LASSO) regression was applied for further selection, and the selected genes were put into multivariate Cox regression analysis to construct a risk model for LUSC. Finally, the CTLIR risk score was calculated based on gene expression and multivariate Cox regression coefficient. The formula is as follows:

$${\text{Risk score }} = \sum\limits_{{\text{i}}}^{{\text{n}}} {E{\text{xpi*}}\beta } {\text{i}}$$

where n, Exp_i_, and β_i_ represent the number of genes, gene expression levels, and regression coefficients, respectively.

### Evaluation of the prognostic value based on the CTLIR risk model

LUSC patients were divided into low- and high-risk groups based on the median risk scores. We utilized the “survival” and “survminer” R packages to draw the Kaplan–Meier survival curve. We performed univariate and multivariate Cox regression analyses to verify whether the CTLIR risk score was an independent prognostic factor for LUSC patients. The results of univariate and multivariate Cox regression were acquired and visualized using the “forestplot” R package. Additionally, we assessed the accuracy of our risk model in predicting prognosis by applying receiver operating characteristic (ROC) curve and calculating the area under the curve (AUC) values.

### Identification of differentially expressed genes (DEGs) and functional enrichment analysis

DEGs were identified by using the “limma” R package for subsequent analysis (*P* < 0.05, |logFC|> 1). Gene set enrichment analysis (GSEA) is a calculation method for determining the statistical significance of the previously defined gene set as well as the concordant heterogeneities [23]. We explored the different biological functions between the low- and high-risk groups annotated using the Gene Ontology (GO) and Kyoto Encyclopedia of Genes and Genomes (KEGG) databases. The GSEA was performed using the “clusterProfiler” R package. False discovery rate value < 0.05 and normalized enriched score > 1 were considered as the significance thresholds.

The gene set variation analysis (GSVA) is an approach for gene set enrichment to unsupervised estimate pathway activity variations among a certain population [24]. We explored the different activities of pathways related to tumorigenesis and progression in the low- and high-risk groups using GSVA. The analysis was realized by the “GSVA” R package, while *P* < 0.05 and |logFC|> 1 were considered statistically significant. The “clusterProfiler” package was used for functional annotation, and the gene set file (c2.cp.kegg.v7.2. symbols.gmt) was obtained from the MSigDB database (https://www.gsea-msigdb.org) [25].

### Correlation analysis of the CTLIR risk score with infiltrating immune cells

The Spearman correlation analysis was used to calculate the correlation coefficient between the CTLIR risk score and 22 types of tumor-infiltrating immune cells. The Estimation of Stromal and Immune cells in Malignant Tumors using Expression data (ESTIMATE) algorithm was used to assess the immune score, stromal score, estimate score, and tumor purity of each LUSC sample [26].

### Analysis of tumor mutation burden (TMB)

The somatic mutation data of LUSC patients in TCGA were downloaded from the Genomic Data Commons Data Portal (https://portal.gdc.cancer.gov/). Somatic mutation data sorted in the form of mutation annotation format were analyzed and then used to calculate TMB using the “maftools” R package [27]. Significantly mutated genes in low- and high-risk groups were analyzed by the “maftools” R package. The difference in mutation frequency between the low- and high-risk groups was evaluated using the Chi-square test, and *P* value < 0.05 was considered significant.

### Prediction of therapeutic response

The immune checkpoint inhibitor immunophenoscore (IPS) profile of LUSC patients was downloaded from the Cancer Immunome Database (TCIA, https://tcia.at/home) [28]. The IPS is a good predictor of the responsiveness to anti-PD-1 and anti-CTLA-4 therapies. In general, a higher IPS indicates a better response to immunotherapy. The IPS is calculated based on representative cell-type gene expression z-scores, with a scale ranging from 0 to 10. In addition, we investigated the predictive capacity of our risk model for the response to chemotherapy and molecular targeted therapy. The 50% inhibiting concentration (IC50) value of the 138 drugs was inferred using the “pRRophetic” R package [29].

### Statistical analysis

Statistical analysis was conducted using R (v4.1.1) software (https://www.r-project.org/). Differences between the two independent groups were evaluated using nonparametric or chi-square tests. The Kaplan–Meier method was used to generate survival curves, and the log-rank test was used to determine statistically significant differences between groups. In our study, the *P* value was two-sided, and *P* < 0.05 was considered statistically significant.

## Results

### CD8^+^ T cell infiltration is related to immunotherapy response

**Fig. 2:**
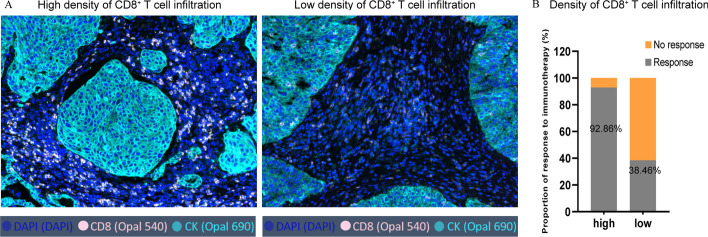
CD8^+^ T cell infiltration is related to immunotherapy response. **A** Representative CD8 staining in tumor tissues of lung squamous cell carcinoma (LUSC) with high or low density of CD8^+^ T cell infiltration. **B** Proportion of response to immunotherapy in the high and low density of CD8^+^ T cell infiltration groups

We collected pretreatment tumor tissue sections from 27 patients with LUSC and analyzed the number of infiltrating CD8^+^ T cells in tumor tissues using mIHC staining. We then divided the LUSC patients into low density and high density of CD8^+^ T cell infiltration groups based on the median number of CD8^+^ T cell counts in the tumor tissues (Fig. 2A and Additional file 7: Table S3). On subsequent treatment with PD-1 inhibitors, LUSC patients were considered responsive to immunotherapy if the progression-free survival (PFS) was longer than 4 months. In contrast, LUSC patients were regarded as having no response to immunotherapy if their PFS did not exceed 4 months. Finally, we found that the proportion of LUSC patients responsive to immunotherapy was higher in the high density of CD8^+^ T cell infiltration group than in the low density of CD8^+^ T cell infiltration group (92.86% vs. 38.46%, *P* < 0.001; Fig. 2B).

### The landscape of infiltrating immune cells in LUSC

**Fig. 3:**
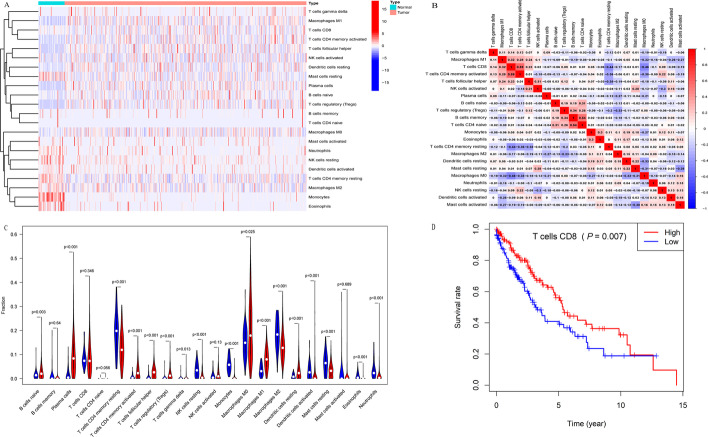
Characteristics of infiltrating immune cells in lung squamous cell carcinoma (LUSC). **A** The heat-map of 22 immune cell abundance in normal and tumor tissues of LUSC. **B** Correlation analysis of all 22 immune infiltrating cells in tumor tissues of LUSC. **C** Differences between the immune infiltrating cells in normal and tumor tissues of LUSC. **D** Survival analysis of infiltrated CD8^+^ T cells in tumor tissues of LUSC

A total of 502 LUSC tumor tissues and 49 adjacent normal tissues in the TCGA database were included to analyze the abundance of infiltrating immune cells. The CIBERSORT algorithm was used to identify the fraction of infiltrating immune cells (n = 22) in LUSC patients (Additional file 8: Table S4). The abundance of immune cell subtypes in each LUSC normal and tumor tissue is shown in the heatmap (Fig. 3A). Overall, high proportions of monocytes and memory resting CD4^+^ T cells were infiltrated in normal tissues, whereas the types of immune cells infiltrated in tumor tissues were diverse. Subsequently, we investigated the correlation between different types of infiltrating immune cells in the tumor tissues. Our results showed that the abundance of CD8^+^ T cells positively correlated with activated memory CD4^+^ T cells (Cor = 0.59), follicular helper T cells (Cor = 0.23), gamma delta T cells (Cor = 0.14) and macrophages M1 (Cor = 0.32) (Fig. 3B). We also found that there were high abundance of CD8^+^ T cells, CD4^+^ memory resting T cells, and macrophages M0/M1/M2 both in normal and tumor tissues (Fig. 3C). Compared to normal tissues, immune cells, including plasma cells (*P* < 0.001), activated memory CD4^+^ T cells (*P* < 0.001), regulatory T (Treg) cells (*P* < 0.001) and macrophages M1 (*P* < 0.001), were more abundant in tumor tissues (Fig. 3C**)**. Although the results showed that the median abundance of CD8^+^ T cells was not significant between normal and tumor tissues (*P* = 0.346), the abundance of CD8^+^ T cell infiltration was significantly different in various tumor tissues (Fig. 3C and Additional file 1: Fig. S1). Moreover, we found that a higher abundance of infiltrating CD8^+^ T cells in tumor tissues was associated with a better prognosis (*P* = 0.007, Fig. 3D). These results indicate the feasibility of risk stratification according to the CTLIR gene signature in LUSC patients.

### Characteristics of the CTLIR risk model

**Fig. 4:**
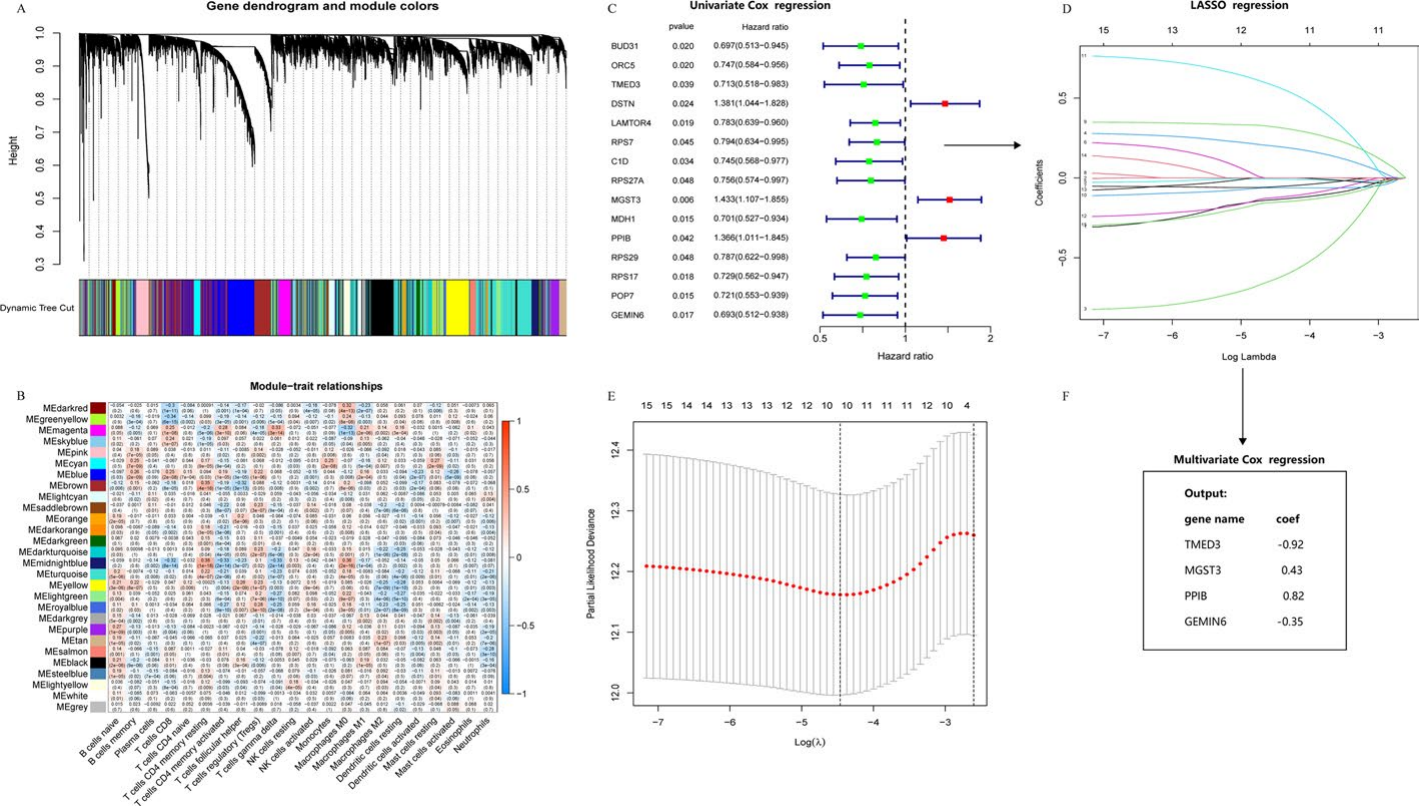
The CD8^+^ T cell infiltration-related gene signature in lung squamous cell carcinoma (LUSC). **A** Cluster dendrogram of the median absolute deviation (MAD) top 5000 genes. **B** Pearson’s correlation coefficients and corresponding *P* value between module eigengenes (ME) and the 22 types of immune cells. **C** Analysis of prognosis-associated genes in the selected module. **D** Least Absolute Shrinkage and Selection Operator (LASSO) coefficient profiles of the 15 prognosis-associated genes. **E** Partial likelihood deviance of variables revealed by the LASSO regression model. The red dots represented the partial likelihood of deviance values, the gray lines represented the standard error (SE), and the two vertical dotted lines on the left and right represented optimal values by minimum criteria and 1-SE criteria, respectively. **F** The identified gene signature and the coefficients by multivariate Cox regression analysis

A total of 502 tumor tissues from LUSC patients were enrolled in the WGCNA analysis. The scale-free network condition was satisfied when β = 5, and the scale independence value achieved 0.9 and lower mean connectivity (Additional file 2: Fig. S2A). Genes with similar expression were grouped into the same module by hierarchical clustering and a height of 0.25 was set as the clipping height threshold. Finally, 28 qualified modules were obtained after merging modules with high similarity (Fig. 4A and Additional file 2: Fig. S2B). We observed that the magenta module had the strongest positive correlation with CD8^+^ T cell infiltration (Cor = 0.25, *P* = 1e−08) (Fig. 4B). Therefore, the magenta module was identified as the most clinically significant module in the subsequent analyses.

We evaluated the prognostic value of genes in the magenta module using univariate Cox regression analysis (Fig. 4C). Fifteen prognosis-associated genes were further subjected to LASSO and multivariate Cox regression analyses. The most appropriate tuning parameter λ for LASSO regression was 0.03 when the partial likelihood binomial deviance reached its minimum value (Fig. 4D, E). Our results showed that the expression of *MGST3*, *TMED3*, *PPIB*, and *GEMIN6* was an independent prognostic factor of LUSC (Fig. 4F). Thus, four genes were included in the CTLIR risk model. Corresponding coefficients were obtained using multivariate Cox regression. Finally, the risk model was established as follows, CTLIR risk score = Exp(MGST3) × 0.43 − Exp(TMED3) × 0.92 + Exp(PPIB) × 0.82 − Exp(GEMIN6) × 0.35.

### Prognosis value of the CTLIR risk model for LUSC

**Fig. 5:**
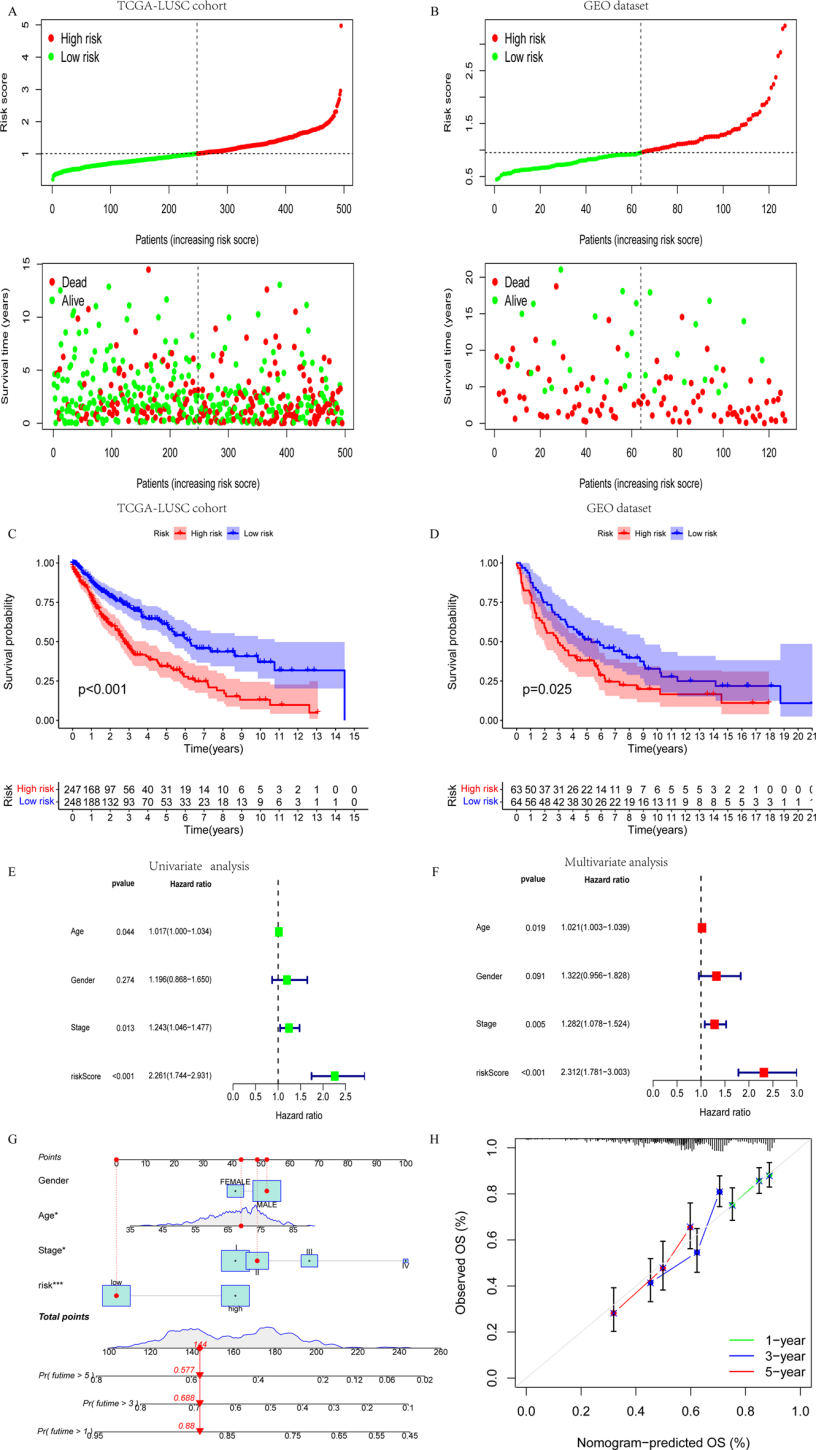
Construction and evaluation of the risk model. **A**, **B** Risk score distribution and survival status of lung squamous cell carcinoma (LUSC) patients from The Cancer Genome Atlas (TCGA)-LUSC cohort and Gene Expression Omnibus (GEO) dataset. **C**, **D** Kaplan–Meier survival analysis of risk score for overall survival (OS) in the TCGA-LUSC cohort and GEO dataset. **E**, **F** Univariate and multivariate analysis of clinical traits and the risk score for OS. **G** Nomogram for predicting the probability of 1-year, 3-year, and 5-year survival in LUSC. **H** Calibration plots of the nomogram

We first ranked the CTLIR risk scores of LUSC patients and analyzed their survival status distribution. Considering the median risk score as the cutoff value, LUSC patients were divided into low- and high-risk groups in both the TCGA-LUSC training cohort and the GEO validation dataset. Our results showed that the proportion of deaths was significantly higher in the high-risk group (Fig. 5A, B). Kaplan–Meier survival curves showed that the OS of LUSC patients in the high-risk group was significantly shorter than that in the low-risk group (log-rank *P* < 0.001, Fig. 5C), and the result was validated in the GEO dataset (log-rank *P* = 0.025, Fig. 5D). Additionally, an ROC analysis was performed to validate the reliability of this risk model. Based on our risk model, the AUC value of 1-year survival was 0.593, which was the highest value when compared with other clinicopathological factors (Additional file 3: Fig. S3A). The AUC values for 3-year and 5-year survival were 0.654, and 0.675, which demonstrated the good predictive accuracy of the risk model (Additional file 3: Fig. S3B).

We also performed univariate and multivariate Cox regression analyses to explore the effect of risk score and other clinicopathological factors on the prognosis ofin LUSC patients. The clinicopathological factors included age, gender, and American Joint Committee on Cancer stage. Both univariate (*P* < 0.001, Fig. 5E) and multivariate Cox regression (*P* < 0.001, Fig. 5F) analyses indicated that the CTLIR risk score was an independent prognostic factor for LUSC patients. Furthermore, we constructed a nomogram generated using the CTLIR risk score and other important clinicopathological traits as a quantitative tool to predict the survival probability of individual LUSC patients (Fig. 5G). Calibration curves revealed ideal consistency of the nomogram in predicting 1-year, 3-year, and 5-year survival rates (Fig. 5H). In summary, these results indicate that our risk model has a certain degree of applicability in predicting the prognosis of LUSC patients.

### GSEA and GSVA between low- and high-risk groups

**Fig. 6:**
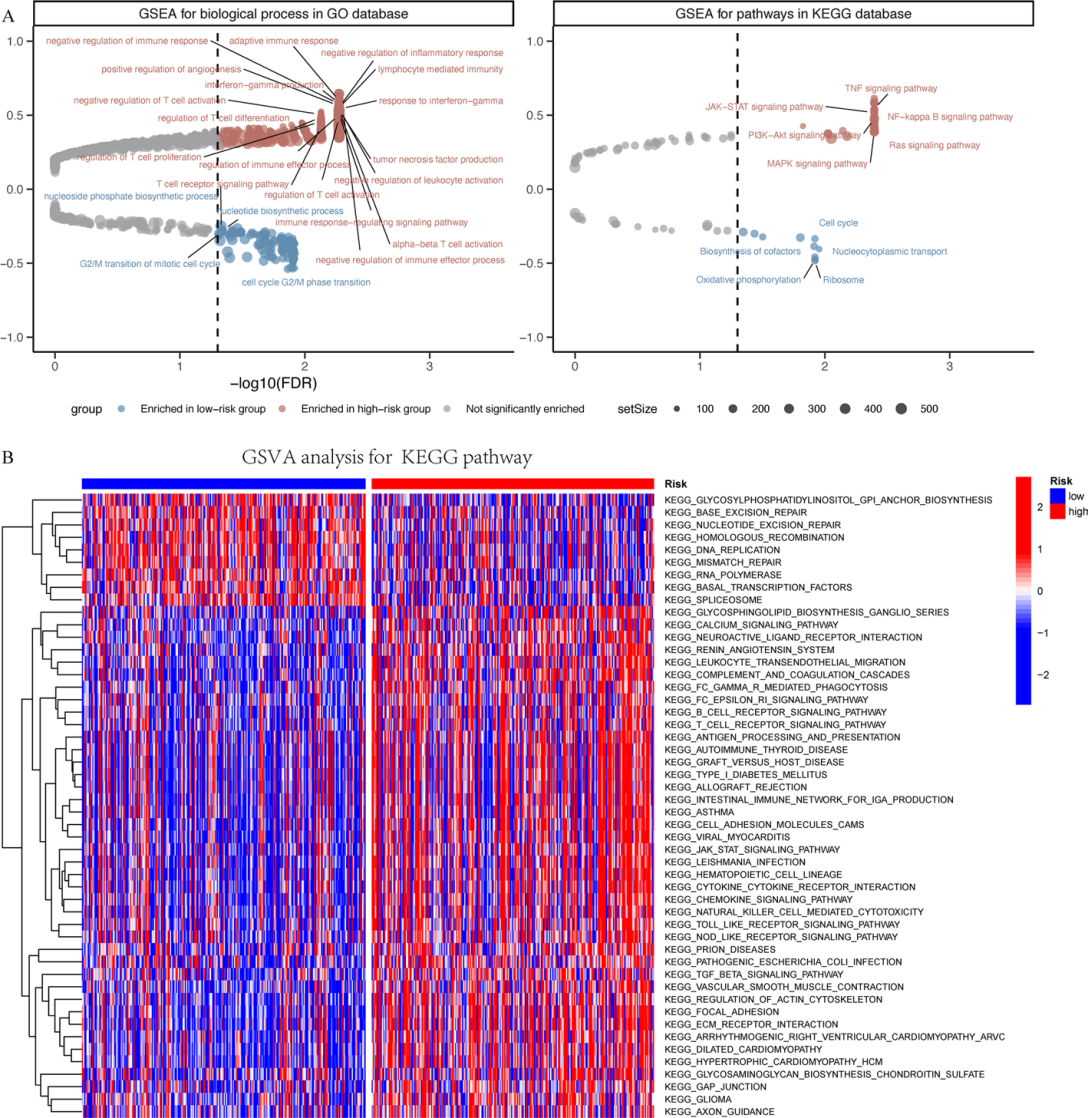
Function enrichment analysis in low- and high-risk groups. **A** Gene set enrichment analysis (GSEA) with annotations of the Gene Ontology (GO) and Kyoto Encyclopedia of Genes and Genomes (KEGG) gene sets. **B** Gene set variation analysis (GSVA) for KEGG pathways

To explore the potential mechanisms leading to different outcomes between the low- and high-risk groups, we performed GSEA with annotations of the GO and KEGG gene sets (Additional file 9: Table S5). According to our results, the gene sets that promote tumor progression, including the JAK-STAT signaling pathway (hsa04630), NF-kappa B signaling pathway (hsa04064), PI3K-Akt signaling pathway (hsa04151), Ras signaling pathway (hsa04014) and MAPK signaling pathway (hsa04010), were significantly enriched in the high-risk group, while the process nucleotide biosynthesis (GO:0009165), regulation of DNA replication (GO:0006275), and cell cycle checkpoint (GO:0000075) were enriched in the low-risk group. Additionally, biological processes associated with the suppression of immune responses, such as negative regulation of immune response (GO:00507770), negative regulation of immune effector processes (GO:0002698), and negative regulation of T cell activation (GO:0050868), were also enriched in the high-risk group (Fig. 6A).

In addition, we conducted GSVA for KEGG pathways based on the differentially expressed genes between the low- and high-risk groups (Additional file 10: Table S6) [30–32]. As shown in our results, the DNA replication and mismatch repair pathways were significantly enriched in the low-risk group. However, many pathways that promote tumor progression, such as JAK/STAT signal pathway and TGF-β signal pathway, were significantly enriched in the high-risk group (Fig. 6B). Based on the above analysis, we characterized the high-risk group as a pro-tumorigenic phenotype.

### Correlations between the CTLIR risk score and infiltrating immune cells

**Fig. 7:**
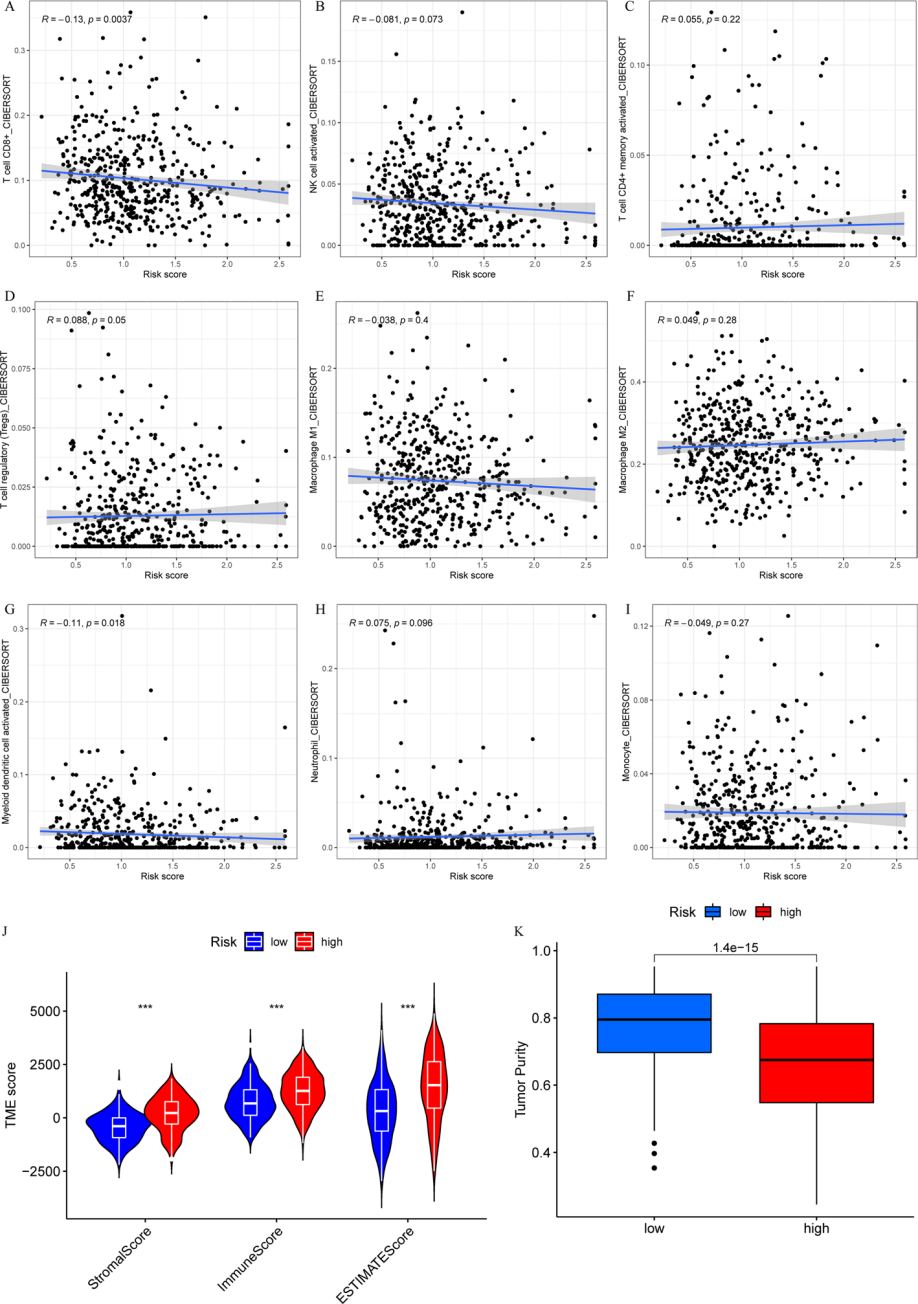
**A**–**I** Correlation analysis between the risk score and infiltrating immune cells. **J** Stromal score, immune score, Estimation of Stromal and Immune cells in Malignant Tumors using the expression data (ESTIMATE) score, and **K** tumor purity in the low- and high-risk groups. ***P < 0.001

To clarify the characteristics of tumor microenvironment (TME), we explored the correlation between the risk score and infiltrating immune cells. Our results indicate that the CTLIR risk score was negatively associated with CD8^+^ T cells (*P* = 0.0037) and activated dendritic cells (*P* = 0.018) and positively associated with Treg cells (*P* = 0.05). However, no correlations were found for other major immune cells, such as activated NK cells (*P* = 0.073), activated memory CD4^+^ T cells (*P* = 0.22), macrophages M1 (*P* = 0.4), macrophages M2 (*P* = 0.28), neutrophils (*P* = 0.096), or monocytes (*P* = 0.27), with the CTLIR risk score (Fig. 7A–I). Compared with the low-risk group, the high-risk group had higher immune, stromal, and ESTIMATE scores (*P* < 0.001; Fig. 7J**)**. Moreover, the tumor purity of the high-risk group was lower than that of the low-risk group (*P* < 0.001; Fig. 7K). Taken together, these results suggested that the TME in the high-risk group tended to have an immunosuppressive phenotype.

### TMB features in low- and high-risk groups

**Fig. 8:**
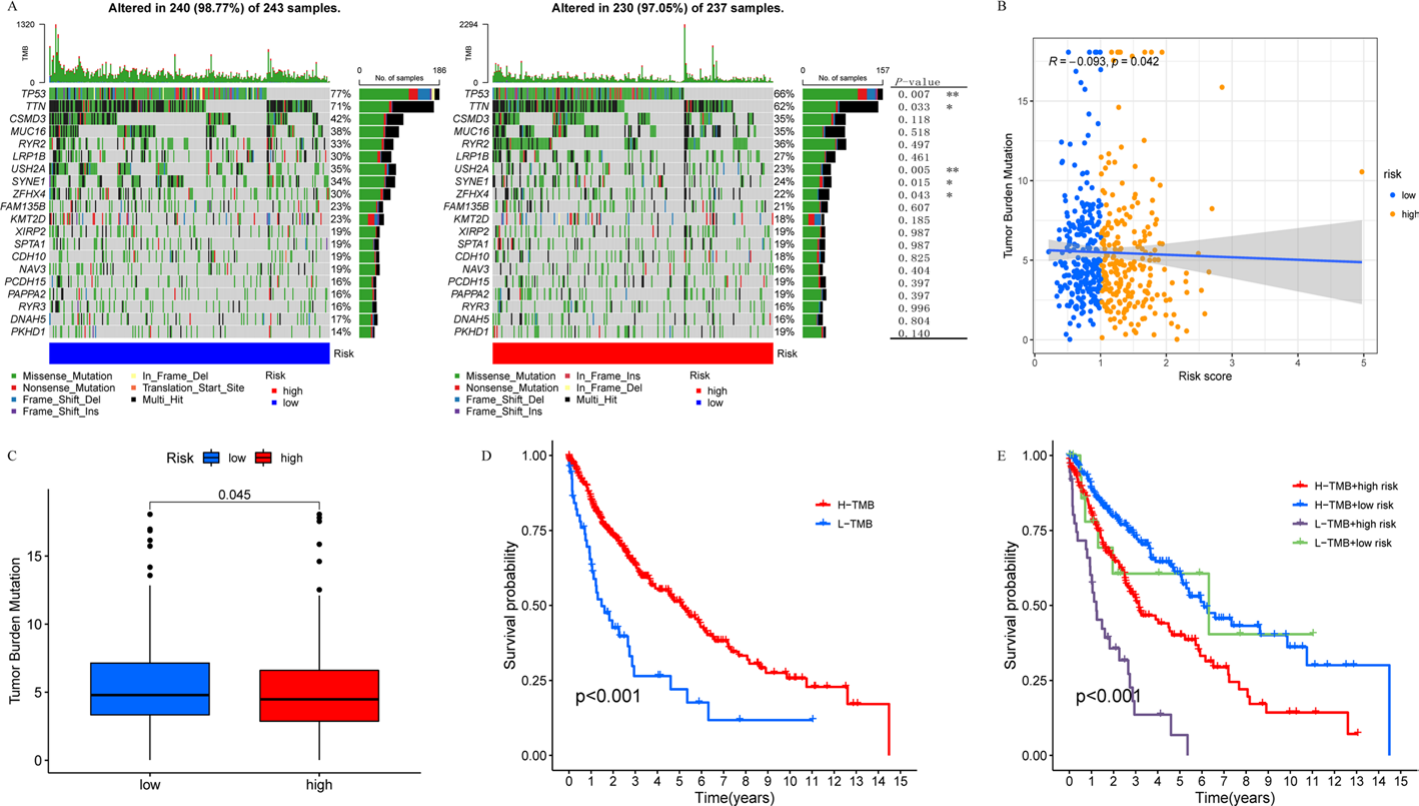
Characteristics of tumor somatic mutation in lung squamous cell carcinoma (LUSC). **A** Waterfall plot of tumor somatic mutation in low- and high-risk groups. **B** Correlation analysis between tumor mutation burden (TMB) and risk score. **C** The TMB level in low- and high-risk groups. **D** Survival analysis of low- and high-TMB groups. **E** Survival analysis stratified by risk score and TMB

To investigate the correlations between the risk score and TMB, we first explored the differences in the somatic mutation status between the high- and low-risk groups. The proportions of mutated samples in the two groups were similar (98.77% vs. 97.05%), with TP53 being the most commonly mutated gene in the two groups. We also observed that the mutation frequencies of genes, including *TP53*, *TTN*, *USH2A*, *SYNE1*, and *ZFHX4* were significantly different between the two groups (*P* < 0.05; Fig. 8A). Furthermore, we found that the risk score was negatively correlated with the TMB levels (Fig. 8B). The TMB was significantly higher in the low-risk group than in the high-risk group (Fig. 8C), and a high TMB was associated with a better survival outcome (Fig. 8D). In the subgroup analysis, we found that the low-risk and high-TMB subgroups had the most favorable survival, while the high-risk and low-TMB subgroups had the worst prognosis (*P* < 0.001; Fig. 8E). Therefore, our study suggests that the low-risk group is likely to be more immunogenic than the high-risk group.

### Significance of the CTLIR risk model in predicting immunotherapy response

**Fig. 9:**
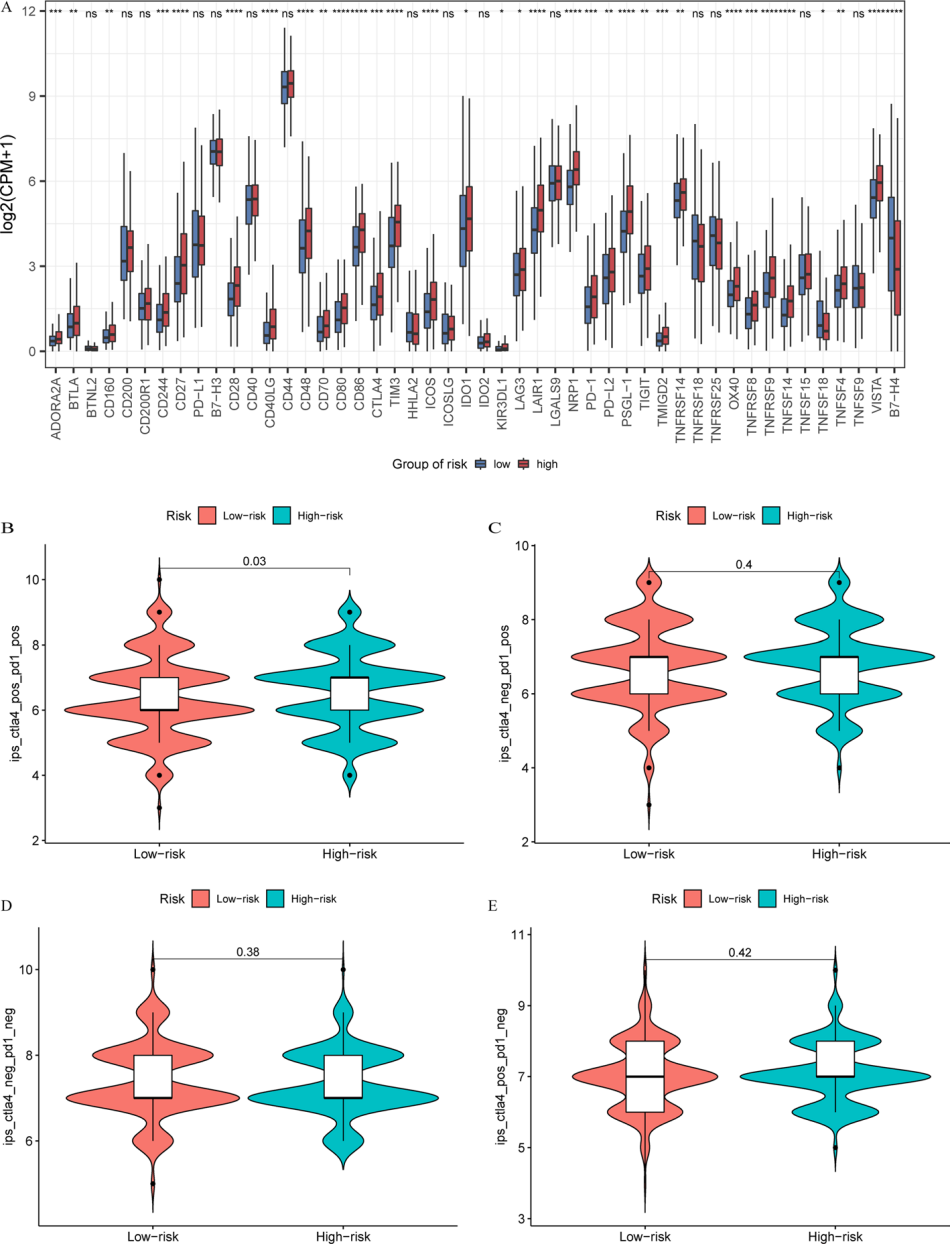
**A** Gene expressions of immune checkpoints in low- and high-risk groups. *P < 0.05; **P < 0.01; ***P < 0.001, ^ns^*P* > 0.05. **B**–**E** The immunophenoscore (IPS) in low- and high-risk groups

To explore the capability of the risk model to predict immunotherapy response in LUSC patients, we compared the gene expression of immune checkpoints in the two groups. Our results showed that the gene expression of 33 immune checkpoints increased in the high-risk group, indicating that immune checkpoint inhibitors may have a pharmacological effect in the high-risk group. It is worth noting that several important immune checkpoints, such as PD-1 and CTLA4, achieved significantly higher expression levels in the high-risk group than in the low-risk group, whereas PD-L1 expression did not differ between the two groups (Fig. 9A).

We obtained the IPS of LUSC patients from the TCIA database to predict their response to immunotherapy (Additional file 11: Table S7). The results showed that the high-risk group had higher IPS-CTLA-4-positive and PD-1-positive scores, which were more sensitive to the combination of CTLA-4 and PD-1 immunotherapy than the low-risk group (*P* = 0.03; Fig. 9B). However, we did not find a difference in the IPS for other types of immunotherapy between the low- and high-risk groups (Fig. 9C–E). Additionally, we explored the differences in drug sensitivity of potential small-molecule compounds for the treatment of LUSC between the low- and high-risk groups and found that the low-risk group was more sensitive to commonly used chemotherapeutic agents and molecular targeted agents, such as etoposide, vinorelbine, erlotinib, and gefitinib (Additional file 4: Fig. S4A–D). In summary, our results indicate that patients with LUSC in the low-risk group were more likely to be sensitive to chemotherapy and targeted therapy, whereas patients in the high-risk group may have a better response to immunotherapy.

## Discussion

LUSC is an especially challenging disease and is associated with a worse prognosis than other histological subtypes of NSCLC [33]. Treatment approaches for NSCLC include surgery, radiotherapy, chemotherapy, immunotherapy, and molecular targeted therapy, either alone or in combination [34]. Immunotherapy, either as a monotherapy or in combination, has become the standard of care in frontline settings for advanced squamous lung cancer [35]. Although ICB can induce long-term remission, most patients fail to achieve a durable clinical response [36]. This has led to considerable effort to identify robust and sensitive risk models for prognosis and ICB response prediction.

Analysis of the tumor-infiltrating immune cell subtypes revealed that CD8^+^ T cells had the strongest positive impact on patients’ survival. The positive prognostic value of CD8^+^ T cells was confirmed in > 18,700 patients across 17 solid cancer types [37]. Tissue microarrays from 335 resected stage I to IIIA NSCLC showed that high densities of CD8^+^ T cells in the stroma were independent positive prognostic indicators for resected NSCLC patients. This results suggest that CD8^+^ T cells mediate a strong anti-tumor immune response in NSCLC [38]. A previous study investigated whether tumor-infiltrating immune cells in biopsy specimens could be used to predict the clinical outcomes of stage IV NSCLC patients. The results showed that patients with more tumor-infiltrating CD8^+^ T cells in the cancer nests had significantly better OS (MST, 388 days vs. 256 days; *P* = 0.007) [39]. However, another study conducted by Wakabayashi et al. indicated that CD4^+^ T cells in the cancer stroma, but not CD8^+^ T cells in cancer cell nests, are associated with a favorable prognosis in NSCLC [40]. It is worth noting that the increased densities of CD8^+^ T cells might be associated with more advanced tumors. Consistently, we found that LUSC patients with a high level of CD8^+^ T cell infiltration had a favorable prognosis.

Next, we identified a gene signature that was highly co-expressed with CD8^+^ T cell infiltration and constructed a risk model to evaluate the prognosis of LUSC patients. In our study, a risk model was established based on four CD8^+^ T cell infiltration-related genes associated with the prognosis ofin LUSC patients, including *TMED3*, *MGST3*, *PPIB*, and *GEMIN6*. According to univariate and multivariate Cox analyses, *TMED3* and *PPIB* had negatively correlated coefficients, and the gene expression level was negatively correlated with the risk score. *MGST3* and *GEMIN6* had positively correlated coefficients, and the gene expression was positively correlated with the risk score. Transmembrane emp24 protein transport domain containing 3 (TMED3) belongs to the TMED family and plays a role in intracellular protein transport [41]. A previous study showed that the expression of TMED3 in tumor cells promotes the progression and development of LUSC [42]. However, we found that high expression of *TMED3* in LUSC tissues was associated with good survival, which indicates that *TMED3* expressed in the TME has a protective effect on survival. Peptidylprolyl cis–trans isomerase B (PPIB) belongs to the cyclophilin family, which participates in protein folding, secretion, and post-translational modification processes [43]. PPIB is associated with malignant progression in gastric cancer, hepatocellular carcinoma, pancreatic cancer, and head and neck squamous cell cancer and is considered a candidate biomarker for these cancers [44]. In our study, LUSC patients with high expression of *PPIB* acquired survival benefits, suggesting that the functions of PPIB may be diverse in different types of cancer. Microsomal glutathione S-transferase 3 (MGST3) is a member of the glutathione S-transferase family, which is involved in detoxification [45]. A previous study showed that overexpression of MGST3 significantly promotes the progression of esophageal squamous cell carcinoma [46]. Gem nuclear organelle-associated protein 6 (GEMIN6) is a component of the survival of the motor neuron (SMN) complex. GEMIN6 is upregulated in LUSC tumor tissues and high expression of GEMIN6 is correlated with poor clinical outcomes [47]. We consistently found that the *MGST3* and *GEMIN6* gene expression is associated with poor survival in LUSC patients.

Based on the risk model, we calculated the CTLIR risk score of patients with LUSC and classified them into low- and high-risk groups with distinct clinical outcomes. Univariate and multivariate Cox analyses revealed that the CTLIR risk score was an independent prognostic factor for LUSC patients. Our results suggest that the LUSC patients in the high-risk group had a worse prognosis than those in the low-risk group. Therefore, we explored the differential molecular mechanisms between low- and high-risk groups. The signaling pathways that promote tumor tumorigenesis and progression, such as JAK-STAT signaling pathway [48], NF-kappa B signaling pathway [49], PI3K-Akt signaling pathway [50], Ras signaling pathway [51], and MAPK signaling pathway [52], were activated in the high-risk group, which may explain why the high-risk group experienced a worse prognosis.

Although the density of immune cell infiltration varies in different tumors, all immune cell components, innate immune cells (macrophages, neutrophils, dendritic cells, innate lymphoid cells, myeloid-derived suppressor cells, and NK cells), and adaptive immune cells (T cells and B cells) are found in the TME [53]. We further explored the association between the CTLIR risk score and immune cells. Our results showed that the CTLIR risk score was negatively correlated with CD8^+^ T cell infiltration but positively correlated with Treg cells. The immunosuppressive microenvironment is an important mechanism by which tumor cells escape immune attacks and promote tumor progression. Immune cells with suppressive phenotypes promote tumor escape. These cell types include tumor-associated macrophages, tumor-associated neutrophils, myeloid-derived suppressor cells, and Treg cells [54]. Treg cells suppress antitumor immunity by inhibiting the killing of tumor cells by antigen-specific CD8^+^ T cells [55]. Moreover, Treg cells orchestrate memory T cell quiescence by suppressing effector and proliferation programs through the inhibitory receptor CTLA-4 [56]. In consistency with the infiltration of immunosuppressive cells in the TME, GSEA also showed that the biological process associated with the suppression of the immune response was enriched in the high-risk group. Hot tumors are usually characterized by extensive CD8^+^ T cell infiltration within the tumor core and at the invasive margin, thereby promoting anti-tumor immunity; cold tumors may show myeloid cell infiltration, but they uniformly show a limited number or absence of CD8^+^ T cells [57]. This indicates that the high-risk group may have characteristics of cold tumors. This tumor profile likely reflects the inefficient generation of anti-tumor immunity, which may lead to a poor prognosis.

Tumors are characterized by a high mutational burden, which is thought to increase the occurrence of tumor-associated antigens, thereby promoting immune recognition by tumor cells. Our results indicated that the CTLIR risk score was negatively correlated with the TMB and that patients with a high TMB had better survival. It is plausible that a higher TMB could act as a prognostic factor for better outcomes, regardless of treatment type. A previous study evaluated the relationship between the TMB and OS in 1415 immunotherapy-naïve patients with diverse advanced malignancies. These results demonstrate that TMB may be a useful prognostic biomarker in immunotherapy-naïve patients, with a protective effect at a higher TMB [58].

Tumor cells can create an immunosuppressive microenvironment by activating immune checkpoint pathways, thus enabling immune escape [59]. Several immune checkpoints have been identified and studied in tumors over recent decades, including but not limited to PD-1 and CTLA-4 [60]. Conventionally, PD-L1 expressed on the surface of tumor cells interacts with PD-1 expressed on the surface of T cells to induce inhibitory signaling [61]. CTLA-4 competes with CD28, a TCR co-stimulatory receptor, to bind ligands, such as CD80 and CD86, which prevents CD28-mediated T cell activation [62]. CD8^+^ T effector cells are thought to be the major type of immune cell affected by the PD-1/PD-L1 checkpoint pathway. In contrast, CTLA-4 predominantly regulates the activity of CD4^+^ T cells, including the effector and Treg cell subtypes [63]. We found the expression of PD-1 and CTLA-4 in the high-risk group was higher than that in the low-risk group. These findings indicate that immunotherapy is a promising strategy for treating high-risk patients with LUSC.

ICB therapy harnesses the immune system to destroy tumor cells by relieving effector cell dysfunction and inhibiting suppressive immune cell populations [64]. Tumors with high expression of PD-L1 or a high TMB may be more sensitive to ICB monotherapy, whereas combination therapy may be required for tumors with fewer infiltrated CD8^+^ T cells and a lower TMB [35]. In our study, we found that LUSC patients in the high-risk group had fewer infiltrating CD8^+^ T cells and a lower TMB than those in the low-risk group. However, high-risk LUSC patients were predicted to have a better response to CTLA-4 blocker in combination with PD-1 blocker. Combining different treatment approaches is a promising strategy for overcoming tumor resistance and sensitizing cold tumors to more effective immunotherapies. As observed in experimental models, combining anti-PD-1 and anti-CTLA-4 monotherapies may result in higher and more durable responses in tumors [65]. Clinical trials have revealed that patients with NSCLC treated with nivolumab plus ipilimumab have a tolerable safety profile and show encouraging clinical activity, characterized by a high response rate and durable response [66, 67]. Based on the above results, we believe that our risk model could be an optional tool for predicting the immunotherapy response in LUSC.

This study had several limitations. First, the stability of our risk model was tested and validated using only GEO datasets. Second, the data were primarily derived from public databases and were retrospective. Prospective cohort studies are required to prove the reliability of our risk model. Moreover, biological experiments should be conducted on the discovered gene signature for in-depth characterization of the mechanisms underlying immune responsive regulation.

## Conclusions

In summary, we comprehensively analyzed the CTLIR gene signature in LUSC and constructed a risk model for LUSC patients to predict prognosis and immunotherapy response. LUSC patients identified as a high-risk group based on our risk model exhibited a worse prognosis and better response to anti-PD-1 in combination with anti-CTLA-4 immunotherapy than the low-risk group. Our research provides new insight for the identification of prognostic biomarkers and prediction of the immunotherapy response in LUSC, which might be helpful for clinical decision-making.

## Supplementary Information

**Additional file 1: Fig. S1.** The bar charts of 22 immune cells in normal and tumor tissues of LUSC.

**Additional file 2: Fig. S2.** The scale independence and clustering of module eigengenes in WGCNA. (A) Analysis of the scale-free network coefficient R-squared for the soft threshold (β) and the mean connectivity for the soft threshold. (B) The hierarchical clustering of gene expressions.

**Additional file 3: Fig. S3.** ROC analysis in LUSC patients. (A) ROC analysis of risk score and clinicopathologic factors. (B) ROC analysis for 1-year, 3-year, and 5-year survival based on the CTLIR risk score.

**Additional file 4: Fig. S4.** The differences in drug sensitivity between low and high-risk groups. (A) etoposide, (B) vinorelbine, (C) erlotinib, (D) gefitinib.

**Additional file 5: Table S1.** The clinical information of LUSC patients from TCGA database.

**Additional file 6: Table S2.** The clinical information of LUSC patients from GEO database.

**Additional file 7: Table S3.** The number of infiltrated CD8^+^ T cells in LUSC tumor tissues.

**Additional file 8: Table S4.** The CIBERSORT results of LUSC patients from TCGA database.

**Additional file 9: Table S5.** GSEA analysis with annotations of GO and KEGG gene sets in low and high-risk groups.

**Additional file 10: Table S6.** GSVA analysis for KEGG pathways in low and high-risk groups.

**Additional file 11: Table S7.** The IPS score of LUSC patients from TCIA database.

## Data Availability

The primary data used to support the findings of this study are included within the supplementary information files. The datasets can be found in Genomic Data Commons Data Portal (https://portal.gdc.cancer.gov/) and the Gene Expression Omnibus (GEO) (GSE30219 and GSE37745, https://www.ncbi.nlm.nih.gov/geo/) databases.

## Abbreviations

LUSC: Lung squamous cell carcinoma
NSCLC: Non-small cell lung cancer
CTLIR: CD8^+^ T cell infiltration-related
mIHC: Multiplex immunohistochemistry
TCGA: The Cancer Genome Atlas
GEO: Gene Expression Omnibus
WGCNA: Weighted correlation network analysis
OS: Overall survival
PFS: Progression-free survival
TME: Tumor microenvironment
ICB: Immune checkpoint blockade
LASSO: Least Absolute Shrinkage and Selection Operator
GSEA: Gene set enrichment analysis
GSVA: Gene set variation analysis
GO: Gene ontology
KEGG: Kyoto Encyclopedia of Genes and Genomes
TMB: Tumor mutation burden
IPS: Immunophenoscore
TCIA: The Cancer Immunome Database
