# Supplementary material for: Comprehensive analyses of a CD8+ T cell infiltration related gene signature with regard to the prediction of prognosis and immunotherapy response in lung squamous cell carcinoma
Source: BMC Bioinformatics. 2023 Jun 6;24:238. doi: 10.1186/s12859-023-05302-3 (PMC10246359; doi:10.1186/s12859-023-05302-3)
Supplement: Supplementary file 1 — Additional file 1: Fig. S1. The bar charts of 22 immune cells in normal and tumor tissues of LUSC. [file 12859_2023_5302_MOESM1_ESM.docx]

**Supplementary Information**

**
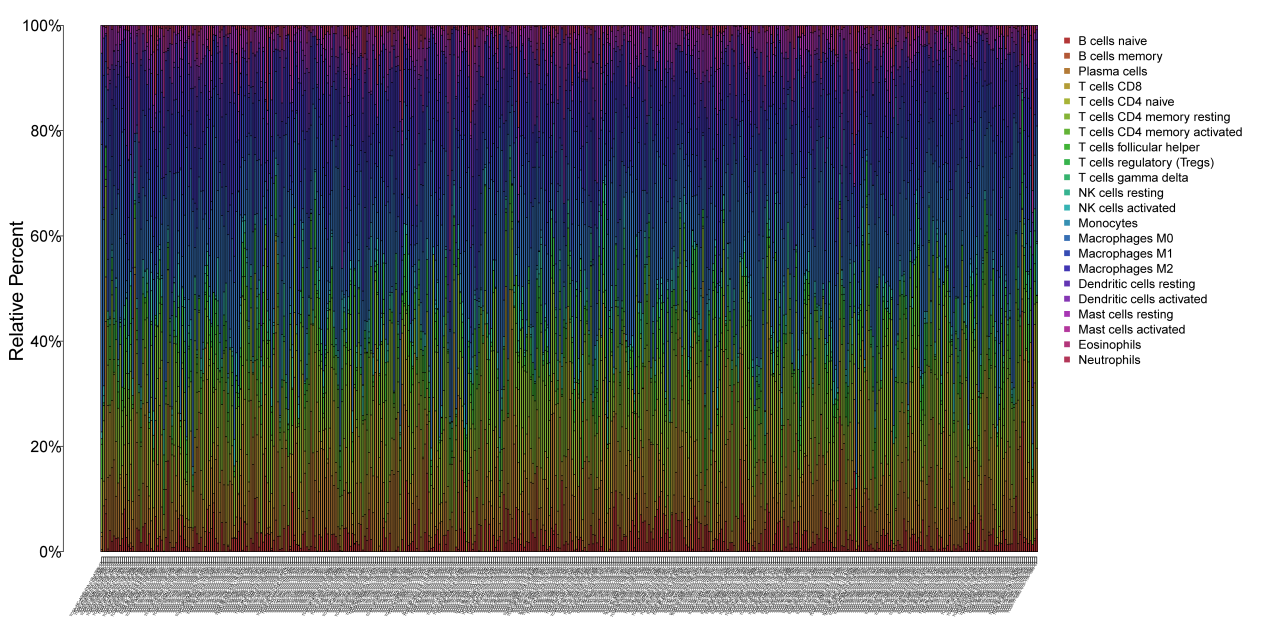
**

Additional file 1: Fig. S1.The bar charts of 22 immune cells in normal and tumor tissues of LUSC.
