# Supplementary material for: Comprehensive analyses of a CD8+ T cell infiltration related gene signature with regard to the prediction of prognosis and immunotherapy response in lung squamous cell carcinoma
Source: BMC Bioinformatics. 2023 Jun 6;24:238. doi: 10.1186/s12859-023-05302-3 (PMC10246359; doi:10.1186/s12859-023-05302-3)
Supplement: Supplementary file 2 — Additional file 2: Fig. S2. The scale independence and clustering of module eigengenes in WGCNA. (A) Analysis of the scale-free network coefficient R-squared for the soft threshold (β) and the mean connectivity for the soft threshold. (B) The hierarchical clustering of gene expressions. [file 12859_2023_5302_MOESM2_ESM.docx]

**Supplementary Information**


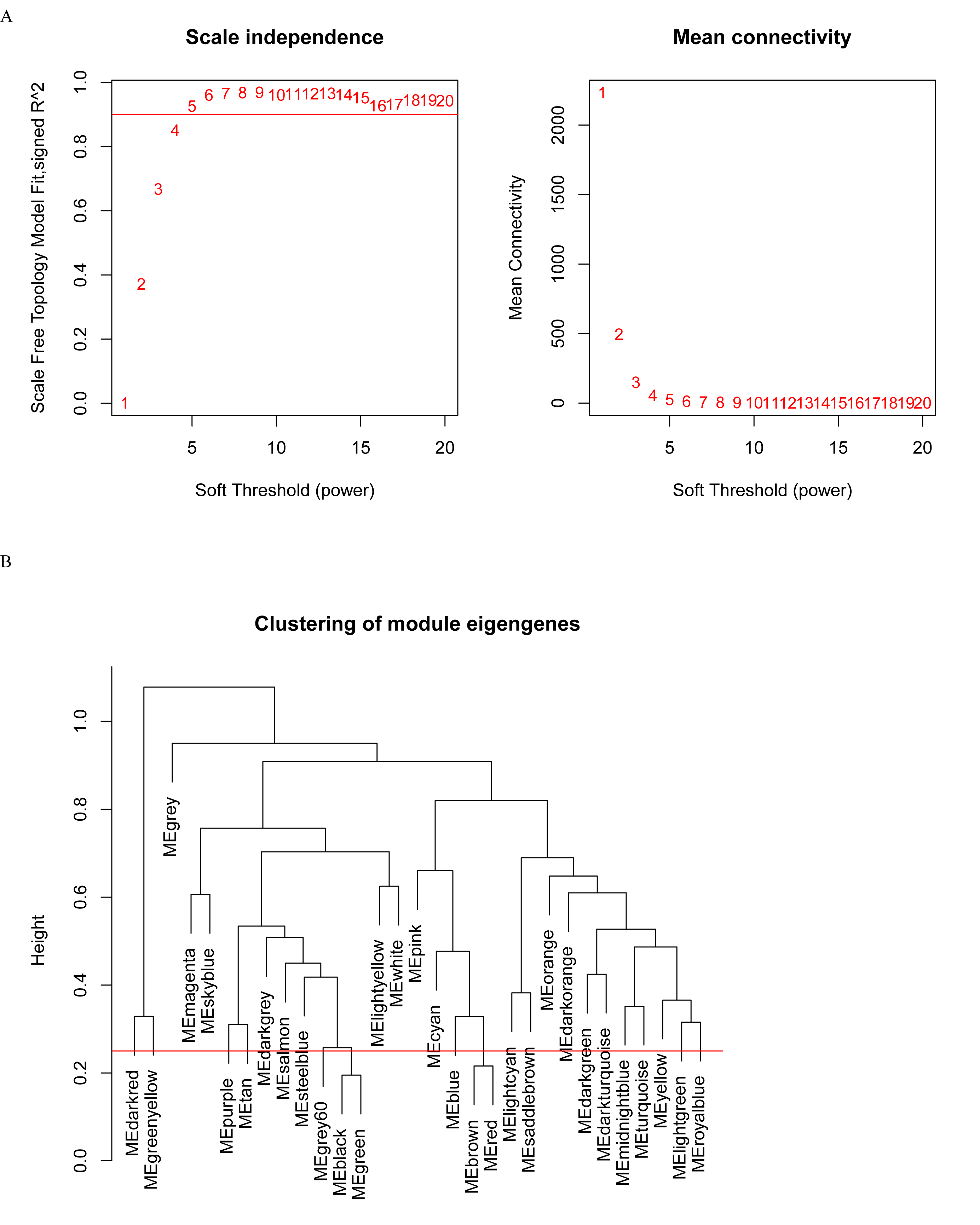


Additional file 2: Fig. S2. The scale independence and clustering of module eigengenes in WGCNA.(A) Analysis of the scale-free network coefficient R-squared for the soft threshold (β) and the mean connectivity for the soft threshold. (B) The hierarchical clustering of gene expressions.
