# Supplementary material for: Comprehensive analyses of a CD8+ T cell infiltration related gene signature with regard to the prediction of prognosis and immunotherapy response in lung squamous cell carcinoma
Source: BMC Bioinformatics. 2023 Jun 6;24:238. doi: 10.1186/s12859-023-05302-3 (PMC10246359; doi:10.1186/s12859-023-05302-3)
Supplement: Supplementary file 3 — Additional file 3: Fig. S3. ROC analysis in LUSC patients. (A) ROC analysis of risk score and clinicopathologic factors. (B) ROC analysis for 1-year, 3-year, and 5-year survival based on the CTLIR risk score. [file 12859_2023_5302_MOESM3_ESM.docx]

**Supplementary Information**


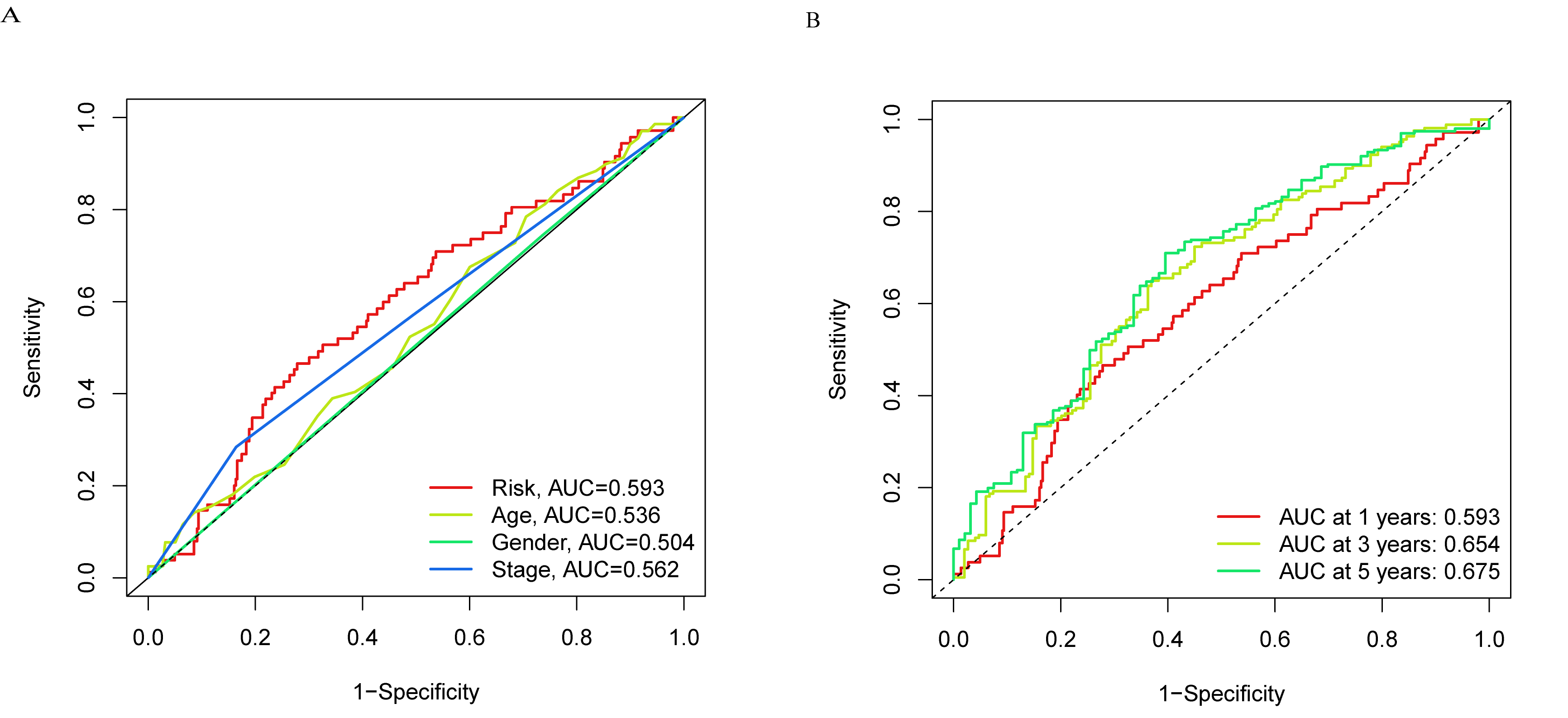


Additional file 3: Fig. S3. ROC analysis in LUSC patients. (A) ROC analysis of risk score and clinicopathologic factors. (B) ROC analysis for 1-year, 3-year, and 5-year survival based on the CTLIR risk score.
