# Supplementary material for: Comprehensive analyses of a CD8+ T cell infiltration related gene signature with regard to the prediction of prognosis and immunotherapy response in lung squamous cell carcinoma
Source: BMC Bioinformatics. 2023 Jun 6;24:238. doi: 10.1186/s12859-023-05302-3 (PMC10246359; doi:10.1186/s12859-023-05302-3)
Supplement: Supplementary file 4 — Additional file 4: Fig. S4. The differences in drug sensitivity between low and high-risk groups. (A) etoposide, (B) vinorelbine, (C) erlotinib, (D) gefitinib. [file 12859_2023_5302_MOESM4_ESM.docx]

**Supplementary Information**


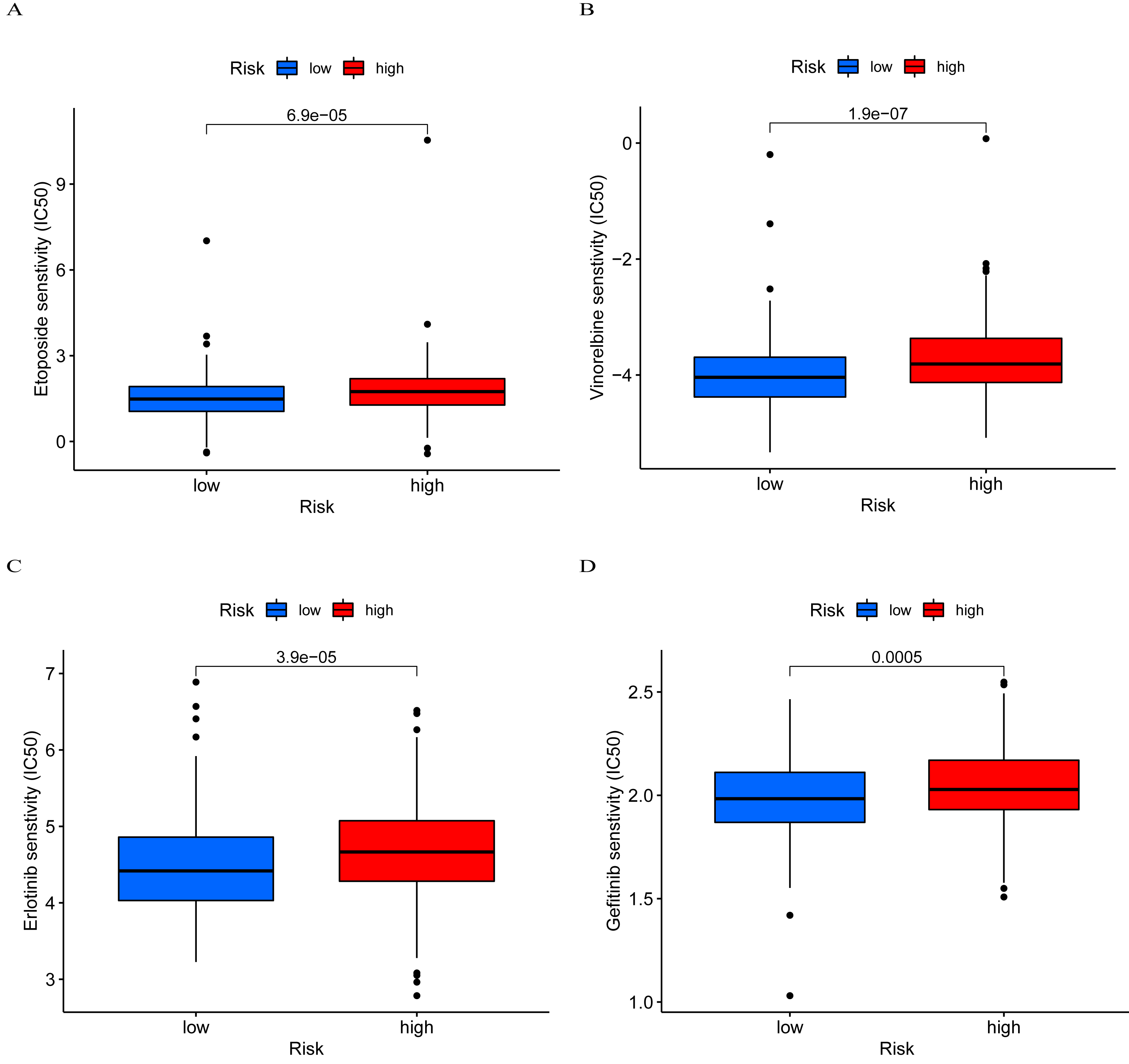


Additional file 4: Fig. S4. The differences in drug sensitivity between low and high-risk groups. (A) etoposide, (B) vinorelbine, (C) erlotinib, (D) gefitinib.
